# Vitamin D and Platelets: A Menacing Duo in COVID-19 and Potential Relation to Bone Remodeling

**DOI:** 10.3390/ijms221810010

**Published:** 2021-09-16

**Authors:** Francesca Salamanna, Melania Maglio, Maria Sartori, Maria Paola Landini, Milena Fini

**Affiliations:** 1 Complex Structure Surgical Sciences and Technologies, IRCCS Istituto Ortopedico Rizzoli, 40136 Bologna, Italy; francesca.salamanna@ior.it (F.S.); maria.sartori@ior.it (M.S.); milena.fini@ior.it (M.F.); 2 Scientific Direction, IRCCS Istituto Ortopedico Rizzoli, 40136 Bologna, Italy; mariapaola.landini@ior.it

**Keywords:** vitamin D, platelets, COVID-19, altered bone remodeling, osteoporosis

## Abstract

Global data correlate severe vitamin D deficiency with COVID-19-associated coagulopathy, further suggesting the presence of a hypercoagulable state in severe COVID-19 patients, which could promote thrombosis in the lungs and in other organs. The feedback loop between COVID-19-associated coagulopathy and vitamin D also involves platelets (PLTs), since vitamin D deficiency stimulates PLT activation and aggregation and increases fibrinolysis and thrombosis. Vitamin D and PLTs share and play specific roles not only in coagulation and thrombosis but also during inflammation, endothelial dysfunction, and immune response. Additionally, another *‘fil rouge’* between vitamin D and PLTs is represented by their role in mineral metabolism and bone health, since vitamin D deficiency, low PLT count, and altered PLT-related parameters are linked to abnormal bone remodeling in certain pathological conditions, such as osteoporosis (OP). Hence, it is possible to speculate that severe COVID-19 patients are characterized by the presence of several predisposing factors to bone fragility and OP that may be monitored to avoid potential complications. Here, we hypothesize different pervasive actions of vitamin D and PLT association in COVID-19, also allowing for potential preliminary information on bone health status during COVID-19 infection.

## 1. Introduction

At the time of writing, the COVID-19 pandemic, due to the respiratory infectious disease caused by severe acute respiratory syndrome coronavirus 2 (SARS-CoV-2), had infected over 225 million people and had caused more than 4.5 million deaths worldwide (https://coronavirus.jhu.edu/map.html, accessed on 3 September 2021). The clinical manifestations of COVID-19 range from asymptomatic to severe forms. A mild spectrum of manifestations with favorable prognosis is present in most patients [1,2]. Nevertheless, generally in elderly patients with several comorbidities, principally diabetes, cardiovascular disease, chronic kidney disease, malignancy, and lung pathologies such as pulmonary fibrosis and chronic obstructive disease, SARS-CoV-2 infection may be complicated by acute respiratory distress syndrome with a high risk of death [1,2]. Severe pulmonary inflammation leads to activation and damage of the pulmonary vasculature and may cause pulmonary thrombosis early in the disease course [3,4,5,6,7]. In an Italian COVID-19 study, the incidence of venous thromboembolism (VTE) (despite thromboprophylaxis) was 27.6% in the intensive care unit (ICU) and 6.6% in the general ward [8]. The rates of ischemic stroke and acute coronary syndrome were 2.5% and 1.1%, respectively [8]. Given these complications and the global spread of COVID-19, SARS-CoV-2 has generated an ever-growing interest both in the mechanisms of infection that lead to disease dissemination and expression, and in the potential risk factors that may have a mechanistic basis for SARS-CoV-2 spread or control. Thus, numerous studies have investigated clinical and laboratory features of COVID-19 patients, as well as inflammatory and organ injury biomarkers [1,2]. Among clinical laboratory indicators, several studies found that disease severity correlates directly with mild thrombocytopenia (a platelet count of 100–150 × 10^9^/L) and altered PLT-related parameters, indicators detected in ~58–95% of severe cases of COVID-19 [9,10,11,12,13,14,15,16,17]. Updated information on COVID-19 disease is continuing to emerge, and several lines of evidence have also suggested that vitamin D deficiency is related to a higher risk of severity of infection [18,19,20,21]. Following a PubMed search for this topic, i.e., ‘COVID-19 AND vitamin D’, more than 500 citations were identified in 2021 (until 13 September 2021) and approximately 372 citations were identified in 2020. For instance, an Israeli study on 7800 individuals found that those with COVID-19 positive test results had significantly lower vitamin D levels compared to those with negative COVID-19 ones [18,19,20,21]. Furthermore, a recent prospective study also showed that vitamin D deficiency is associated with higher blood glucose levels and inflammatory markers, as well as to lower levels of serum calcium, albumin, PLTs and lymphocytes, in deceased COVID-19 patients [22]. All these alterations further reflect the hypercoagulable state present in severe COVID-19 patients, which could promote thrombosis in the lungs and in other organs [11,12,13,14,15,16,17,18,19,20,21,22,23,24,25,26,27,28]. In fact, vitamin D, in addition to its traditional role in mineral metabolism/skeletal health, has anti-inflammatory and antithrombotic effects [29]. Mohammad et al. described the role of vitamin D in inflammatory and coagulation pathways, as well as its role in endothelial activation [30]. The feedback loop linking inflammation, coagulation, and vitamin D also involves PLTs, the plasma coagulation cascade, physiologic anticoagulants, fibrinolytic activity, and proinflammatory cytokines and chemokines. These mediators also lead to overactivation, aggregation, and retention of PLTs, as well as the formation of thrombus at the injured site, which may cause PLTs and megakaryocytes to deplete, resulting in decreased PLT production and increased consumption [25]. In addition to the roles of vitamin D and PLTs in inflammation, thrombosis, and endothelial dysfunction, they could play a critical role in immune response [31]. Thus, vitamin D and PLTs seem to share numerous roles in different physiological and pathophysiological processes. Another *‘fil rouge’* linking vitamin D and PLTs is represented by their role in mineral metabolism and skeletal diseases, where vitamin D deficiency, low PLT count, and altered PLT-related parameters are linked to altered bone remodeling due to osteoporosis (OP) [21,27,28,32,33]. In this context, it is interesting to point out that severe COVID-19 patients are also characterized by the presence of several predisposing factors to bone fragility [34,35,36,37], which are the high expression of proinflammatory cytokines, high hypocalcemia rate, several comorbidities such as diabetes, treatments with high doses of glucocorticoid, vitamin D deficiency, thrombocytopenia, and PTL activation [1,2,38,39,40,41,42]. However, outside of these findings, no clinical data yet exist on the real link between bone fragility and COVID-19 infection. It is plausible that this potential link will emerge in the coming months as it did for the SARS epidemic where reports of arthralgia, reduced bone mineral density (BMD), and osteonecrosis were initially attributed to the high dosages of steroids used to treat the infection. In fact, only after several years was it shown that SARS infection could lead to an alteration of bone metabolism independently of the drugs used to treat the infection [43].

Considering all the shared roles of vitamin D and PLTs in coagulation, thrombosis, inflammation, endothelial dysfunction, immune response, and OP condition, here, we hypothesize different pervasive actions of the vitamin D and PLT association in COVID-19, also allowing for potential preliminary information on bone health status in COVID-19 infection. This overview will provide researchers and clinicians with a potential starting point for linking vitamin D deficiency and PLT levels to COVID-19, while also allowing for prospective preliminary information on bone health status during COVID-19 infection.

## 2. Vitamin D

Vitamin D, usually evaluated through the dosage of the active metabolite hepatically hydroxylated, 25-hydroxyvitamin D (25OHD), is a fat-soluble vitamin, mainly produced during exposure to sunlight and partially taken via the diet, which plays a key role in calcium homeostasis. Its activity can be mediated by both genomic and nongenomic actions via interplay with the vitamin D receptor (VDR), modulating the expression of several genes, including some involved in immune response [44]. Vitamin D is able to modulate both proinflammatory cytokines (tumor necrosis factor-α (TNF-α) and interferon-γ (IFN-γ)) and anti-inflammatory cytokines (interleukin-10 (IL-10)), promote the production of antimicrobial peptides (human cathelicidin, LL-37, and defensins), and induce the expression of genes related to cellular junctions, thereby preserving the integrity of the epithelium [45].

The possibility of identifying a correlation between COVID-19 infection and vitamin D arises from the evidence that negative fluctuation of vitamin D levels is associated with the onset of cytokine storm, increased risks of arterial and venous cardiovascular events, and higher susceptibility to viral and bacterial infective diseases. Such evidence has also been observed in COVID-19 patients [46,47]. The actual correlation between vitamin D levels and the severity of COVID-19 infection is a topic of debate, despite several observations to this effect. Many retrospective clinical studies have highlighted the consistency of a negative correlation between vitamin D levels and COVID-19 infection, statistically significant in many European countries (*p* = 0.033). A retrospective observational analysis of 191,779 patients highlighted a higher positivity rate of SARS-CoV-2 infection in subjects with vitamin D deficiency, identifying a strong correlation between SARS-CoV-2 positivity and circulating 25(OH)D levels, independent of gender, age, ethnicity, or environmental influences [48]. The same association was found in another study that also highlighted a link between 25(OH)D levels and lymphocyte percentage count and C-reactive protein (CRP) levels in COVID-19 patients. The intuitive relapse of this correlation on the course of the disease seems to be validated by the lower percentage of fatal outcomes in patients with ‘sufficient’ levels of vitamin D in comparison to those with lower parameters (9.7% vs. 20%). [49] However, other studies did not find a strong correlation between low levels of vitamin D and increased severity or higher risk of fatal outcome in COVID-19 patients. [50] Certainly, the fact that vitamin D deficiency is a very frequent condition in the world population may have led to this aspect being little considered. It is estimated, in fact, that almost half of the European population does not receive an adequate intake of vitamin D and, interestingly, this aspect can be seen reflected in the higher incidence of SARS-CoV-2 infection in Europe [20,51]. Surely, an impairment of the vitamin D levels is a negative prognostic factor for infective diseases, especially viral ones [20,51]. With reference to COVID-19 infection, a concomitant vitamin D deficiency seems to worsen some risk factors, such as old age or belonging to ethnic groups with little exposure to the sun and comorbidities such as obesity or cardiocirculatory imbalances. As for gender, it is now clear that the male gender is a predisposing factor for SARS-CoV-2 infection, and this evidence may be correlated with the different gender-related expression of ACE2 and TMPRSS2, which are both involved in the viral mechanism of SARS-CoV-2, in addition to the stronger immune response of the female gender. The discordant data emerging from literature do not allow establishing a gender correlation between vitamin D and COVID-19 infection onset or worsening. However, some researchers have highlighted the gender-related activity of vitamin D3 in autoimmune disease. In this context, vitamin D3 seems to be influenced by estrogen levels, leading to a more aggressive inflammatory response and cytokine activity in females. [52] Nevertheless, analyses of randomized controlled trials, as well as causal modeling studies and analyses of variance, highlight the biological plausibility of a correlation between low vitamin D levels and aggressiveness of COVID-19 disease, influencing viral replication and development of the “cytokine storm” [53]. Vitamin D seems to also play a role in the manifestation of other serious COVID-19-related symptoms such as those linked to cardiovascular and hemostasis systems [47]. In this context, a recent prospective cohort study on patients with severe vitamin D deficiency demonstrated that supplementation with high-dose cholecalciferol (vitamin D_3_) was linked with reduced thrombin generation and decreased clot density, without a change in fibrinolytic times, suggesting that vitamin D supplementation decreases the prothrombotic profile [47]. Thus, the increased thrombotic risk in COVID-19 infection could also be linked to hypovitaminosis D; however, to date, there have been no intervention studies with vitamin D. In addition, the involvement of vitamin D in endothelial repair and in the inhibition of transformation of macrophages in foam cells, thus preventing the development of atherosclerosis, is also well known. These mechanisms are in a delicate balance, which can be impaired by significant increases and decreases in vitamin D levels. Intriguingly, the correlation between vitamin D status and severity of COVID-19 infection goes through the renin–angiotensin–aldosterone system (RAAS) [54]. In fact, preclinical studies have shown a mechanism of overexpression of the RAAS system related to vitamin D receptor depletion, with a consequent increase in thrombogenic events. Therefore, an impairment of vitamin D levels might further negatively influence the course of COVID-19 disease, whose infection exploits the ACE2 receptor, which is a known key player of the RAAS system [55]. On the other hand, since COVID-19 is an infectious disease principally affecting the pulmonary system, vitamin D deficiency in itself represents a worsening factor. Historically, supplementation with vitamin D, directly with diet or indirectly via encouraging exposure to sunshine, has been employed as a therapeutic strategy for many lung infections, such as tuberculosis, and, in recent years, many clinical studies have shown its beneficial effects in association with standard pharmacological therapy [56]. A genomic study highlighted the presence of 108 differentially expressed host genes (DEHGs) in SARS-CoV-2-infected normal human bronchial epithelial (NHBE) cells. Exploiting bioinformatic and systems biology tools, a “*host response signature network*” was identified and associated with pathways involved both in inflammatory response and in nongenomic vitamin D activity. Considering the involvement of vitamin D in immune response via inhibition of the TNF-induced NF-κB1 signaling pathway and activation of the IFN-α-induced Jak–STAT signaling pathway, these findings indicate of the promise of combining vitamin D and INF-α in counteracting COVID-19 disease [57]. The possible efficacy of vitamin D supplementation in the therapeutic plans of patients affected by COVID-19 disease exploits the effects on both adaptive and innate immunity. The antiviral effect does not act directly on viral load or replication but acts on the “cytokine storm” via the VDR and CYP27B1, the enzyme responsible for the conversion of vitamin D into calcitriol, its active metabolite. This action leads to a negative modulation of IL-6, IL-8, TNF-α, IFN-β, macrophage chemotactic protein-1, type 1 interferon, TNF-α, and reactive oxygen species, thus mitigating the effects of COVID-19 infection [58]. Some trials are also exploring the use of dexamethasone which, by modulating the immune system, seems to have beneficial effects in preventing the most acute symptoms of COVID-19 infection, such as the development of acute respiratory distress syndrome (RDS) and critical pneumonia, while avoiding the use of invasive artificial ventilation systems. Dexamethasone administration seems to positively regulate vitamin D levels and transcription of the VDR gene, while also reducing the CRP rate, thus preventing a fatal outcome [59]. These ongoing trials provide controversial results and many open issues, such as the correct dosage of vitamin D to administer, in terms of both amount and frequency, and the appropriate pharmacological formulation (vitamin D, active vitamin D, or analogues). The great variety of choices in these aspects, together with the heterogeneity of treated patients for previous clinical history, presence of comorbidities, and presentation of coronavirus disease, makes it difficult to analyze data collected so far and to establish a consensus [60]. On the other hand, vitamin D toxicity is quite a rare occurrence unlike the pathological conditions related to an inadequate vitamin D supply, and this might lead some authors to consider that the benefits of supplementation outweigh the disadvantages [61,62]. Such misleading feedback has also led to a distrustful view of vitamin D supplementation by some clinicians in the treatment of COVID-19 patients. An aspect that generates concern is that supplementation with vitamin D, while mitigating the more severe effects and symptoms of COVID-19, may nevertheless make patients more susceptible to disease recurrence and secondary lung infections, thus undermining the effects of the therapy [63]. Other findings that should be considered are suggested by data collected from patients, which indicate a lowering of 25(OH)D levels dependent on system infection, focusing on the possibility that the low vitamin D level is a consequence of and not a contributing cause to the infection [64].

**Figure 1:**
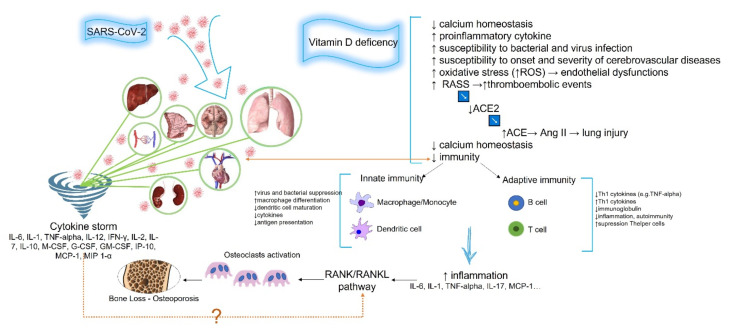
Graphical overview of vitamin D deficiency and its potential relationship with SARS-CoV-2 infection and altered bone remodeling due to osteoporosis. Angiotensin converting enzyme 2 (ACE2), renin–angiotensin–aldosterone system (RASS), reactive oxygen species (ROS), angiotensin (Ang) II, IL-6, interleukin (IL)-1, tumor necrosis factor (TNF)-alpha, IL-12, interferon (IFN)-γ, IL-2, IL-7, IL-10, IL-17, macrophage-colony stimulating factor (M-CSF), granulocyte colony-stimulating factor (G-CSF), granulocyte-macrophage colony-stimulating factor (GM-CSF), C–X–C motif chemokine ligand 10 (IP-10), monocyte chemoattractant protein-1 (MCP-1), macrophage inflammatory protein 1 (MIP 1)-α, receptor activator of NF-kappa B/receptor activator of NF-kappa B ligand (RANK/RANKL).

The importance of integrating treatment plans with vitamin D supplementation has also been highlighted for countering and mitigating the effect of the “cytokine storm*”* on bone tissue health [65]. The higher expression of inflammatory cytokines, such as IL-6 and IL-1, induces an overactivation of the RANK/RANKL pathway, leading to an unbalanced bone resorption and OP risk [66]. In fact, IL-6 promotes RANKL upregulation in osteoblastic cells. IL-1 may not only stimulate osteoclast generation, but also seemingly promote mature osteoclasts to perform more resorption cycles via RANKL activity modulation. IL-1 is further implicated in bone metabolism as an osteoblast activator; osteoblasts secrete RANKL, which stimulates survival and differentiation of the osteoclast precursors to mature osteoclasts through RANK. IL-1 and IL-6 also directly enhance osteoclast activity via RANKL-independent mechanisms [67]. Evidence from both epidemiological and observational studies has shown that the immunoregulatory mechanisms of vitamin D may modulate the effect of these cytokines on bone health and subsequent fracture risk [68]. It is this ability of vitamin D to suppress cytokine production that motivated our focus on vitamin D deficiency and its association with severe COVID-19 and altered bone remodeling due to OP. In fact, recent data indicate a correlation between vitamin D treatment for OP and COVID-19 incidence, with beneficial effects for patients already undergoing such therapy. Blanch-Rubiò et al. showed that, in a cross-sectional study on 2012 patients affected by OP or fibromyalgia, two-thirds of those treated with vitamin D and calcium supplements had reduced risk of contracting COVID-19 [69]. This might suggest that patients receiving adequate treatment, including vitamin D, for already diagnosed bone metabolism alterations, could benefit from a protective effect against SARS-CoV-2 infection and a preventive effect on possible related bone sequelae. This implies that, on the other hand, patients for whom the diagnosis and/or treatment of impaired bone remodeling is less frequent are more exposed to the risks associated with the disease. For example, there is growing recognition of the underestimation of OP in male patients. Recent data highlight not only a great incidence of this pathology in males but also a higher risk of complications, such as fractures [70]. Considering the higher incidence of SARS-CoV-2 infection in men, it could be interesting to observe the infection bone effects in the male age group at higher risk for OP. This might also be true for other categories, for which OP is underdiagnosed and consequently undertreated, such as patients suffering from rare diseases leading to bone metabolism alteration [71]. Furthermore, it might be worth assuming that subjects with borderline BMD index, both men and women, as well as those already suffering from vitamin D deficiency, such as elderly people [72], may be more at risk of rapidly developing an altered bone remodeling pathology and, consequently, more prone to SARS-CoV-2 infection, due to the combined effect of pre-existing low vitamin D levels, not compensated for by adequate treatment, as well as more prone to the effects of the *“cytokine storm”* of infection, further exacerbated by vitamin D imbalance (Figure 1).

## 3. Platelets

PLTs are complex anucleate cells containing different types of granules, i.e., α-granules, dense or δ-granules, and lysosomes [73,74,75,76]. Present throughout the vascular system, PLTs replicate signals from the endothelium, circulating cells, or other blood components [77]. The vascular relevance of PLTs is due to their key role in thrombosis and in intervening in myocardial infarction, stroke, and VTE [74,76,77,78,79]. In addition to their hemostatic function, PLTs actively participate in the immune response [75,80]. Even though the immune role of PLTs is not yet fully understood, there is a delicate balance between its pathogenic response and its regulation of thrombotic and hemostatic functions [75,80]. PLTs also interact with several types of viruses that are often associated with thrombocytopenia and, in some cases, also with thrombosis [81]. Thrombocytopenia is a simple and readily available biomarker related to infection severity and risk of mortality in the ICU. Furthermore, thrombocytopenia is also associated with higher disease severity scores such as Multiple Organ Dysfunction Score (MODS), Simplified Acute Physiology Score (SAPS) II, and Acute Physiology and Chronic Health Evaluation (APACHE) II [82]. 

Severe forms of COVID-19 are associated with a high thrombosis incidence and a high percentage of pulmonary and systemic PLT-rich microthrombi that may finally progress to overt disseminated intravascular coagulation [83,84,85,86]. Numerous studies have reported a moderate thrombocytopenia and an increase in PLT activation, PLT reactivity, and PLT–leukocyte aggregates in COVID-19 patients in comparison to heathy controls [87,88,89,90]. A temporal trend of lowering PLT counts in COVID-19 patients could indicate a worsening thrombotic state [87,88,89,90]. It was reported that a PTL count <200 × 10^9^/L at admission was related to a threefold higher mortality rate [91]. On the other hand, improvement of thrombocytopenia in COVID-19 patients can indicate clinical progress [91]. It was also reported that patients with severe COVID-19 displayed a higher degree of PTs activation, PLT–monocyte aggregation, and PLT–lymphocyte ratio (PLR) [88,92,93,94]. In a recent case–control study, a lower PLT count in COVID-19 patients was also associated with a higher mean PLT volume (MPV), PLT distribution width (PDW), and PLT large cell ratio [95]. It was also reported that MPV differences between the first and third days of hospitalization were significant parameters in patients with COVID-19 in predicting mortality, and a one unit increase in MPV led to a 1.76-fold increase in the mortality rate [96,97,98]. However, it was shown that this trend toward greater MPV persists even in COVID-19 patients without thrombocytopenia. The increased MPV suggests an increase in circulating young PLTs as the body’s response to thrombocytopenia. A significant trend of elevated immature PLT fraction (IPF) (or reticulated PLTs) was also detected in COVID-19 patients [99]. Since immature PLTs are more functional, this aspect could lead to an increased number of clotting events in COVID-19 patients [100]. In addition to having increased numbers of immature PLTs, COVID-19 patients also have increased levels of circulating activated PLTs, as confirmed by the high level of P-selectin on their surface membranes [87]. Although all these data are preliminary and should be confirmed in a larger study population, they provide a scientific rationale to support the design of clinical trials aiming to assess whether the use of anti-PLT agents may mitigate and/or reduce the coagulopathy occurring in COVID-19 patients. Numerous trials, e.g., NCT04324463, NCT04333407, NCT04409834, NCT04518735, NCT04320615, NCT04349410, NCT04479358, and NCT04412772, are now ongoing with the final goal to noticeably improve a patient’s overall prognosis. A key point would be to understand the potential reasons for thrombocytopenia, increased PLT reactivity, and altered PLT-related parameters in COVID-19 patients that are likely multifactorial and could include a direct effect of SARS-CoV-2 on PLT production, autoimmune destruction of PLTs, or increased PLT consumption. According to information on previous infections such as SARS, it was proposed that the combination of viral infection and mechanical ventilation involves endothelial damage that leads to PLTs activation, aggregation, and thrombosis in the lung, which dramatically increases PLT consumption [101]. Additionally, as the lung can be a PLT release site from fully mature megakaryocytes, a decrease in or morphological alternation of the pulmonary capillary bed can lead to unbalanced PLT defragmentation [101]. Coronaviruses can also promptly infect elements of the bone marrow, leading to an abnormal hematopoiesis or to an autoimmune response against blood cells [101,102]. Lastly, persistent low-grade disseminated intravascular coagulation may also lead to a low PLT count in SARS [101]. However, since the pathophysiologic mechanisms behind each infection are different, it should be underlined that numerous differences can be observed between SARS and COVID-19 [102,103]. In fact, Zhang et al. evaluating the specific role of PLTs in COVID-19 infection, showed that ACE2 and TMPRSS2 are expressed in PLTs, and that SARS-CoV-2 directly triggers PLTs and strengthens their prothrombotic role and inflammatory response via spike/ACE2 interactions [104]. This study found a novel function of SARS-CoV-2 in PLT activation, showing that SARS-CoV-2-induced PLT activation may directly participate in thrombus formation and inflammatory responses in COVID-19 patients via the binding of spike to ACE2.

**Figure 2:**
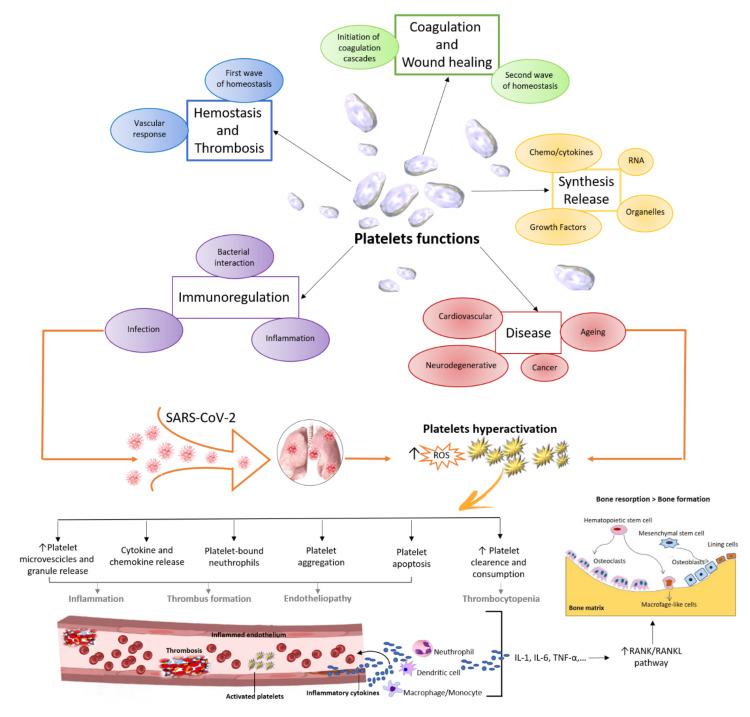
Graphical overview of platelet functions and activities, as well as their potential relationship with SARS-CoV-2 and altered bone remodeling due to osteoporosis. Reactive oxygen species (ROS), interleukin (IL)-1, IL-6, tumor necrosis factor (TNF)-alpha, NF-kappa B/receptor activator of NF-kappa B ligand (RANK/RANKL).

The thrombocytopenia altered PLT-related parameters, and increased PLT reactivity present during COVID-19 infection were also previously associated with low bone mineral density (BMD) due to OP, both in women and in men [105,106,107,108,109,110,111]. A recent study showed that high PLT counts were related to low BMD at all sites in a Sweden cohort of OP men (69–81 years) [111]. Aging is a well-known risk factor for OP, as well as for COVID-19, and a mild thrombocytopenia and altered PLT-related parameters, such as MPV and PDW, were also found to increase with aging [112]. Furthermore, megakaryocytes, responsible for PLT production and related to PLT number and size, increase in the bone marrow with aging, leading to an imbalance between osteoblastic and osteoclastic functions that in turn lead to increased production of proinflammatory cytokines such as IL-1, IL-6, and/or TNF-α (‘inflammaging*’*) [113,114]. These cytokines, also produced in high amounts during COVID-19 and OP, play a key role in osteoclastogenesis, enhancing the bone resorptive capacity via RANK/RANKL/OPG signaling. This system is the central and primary regulator of bone remodeling, but it is not the only involved mechanism. An emerging role in bone pathophysiology has also been attributed to the immune system, specifically to T cells and B cells that are known to contribute to OP pathogenesis [115]. Similarly, under SARS-CoV2 infection, the immune system is damaged with a reduction in lymphocytes in peripheral blood, mainly T cells and B cells [116]. In these situations, T cells were activated and differentiated into Th17 cells that produced IL-17, which in turn stimulated RANK expression, thus accelerating osteoclast differentiation and leading to bone destruction [117]. IL-17 also augments local inflammation. Consequently, the production of inflammatory cytokines such as TNF and IL-6 is increased, thereby strengthening RANKL expression and indirectly promoting osteoclastogenesis [117]. Thus, both COVID-19 and OP seem to share an immune and inflammatory involvement. This shared contribution has been reported for several pathological conditions, some of which are also considered risk factors for more severe forms of COVID-19, i.e., diabetes, immune thrombocytopenia, and Kawasaki disease. In these pathological conditions, T- and B-cell components have been linked with increased PLT activation and surface expression of CD154, a PLT-soluble mediator critical to the initiation and propagation of the adaptive immune response [118,119]. In conclusion, all these data suggest that PLTs share broad and multifaced functions in both COVID-19 and OP [120] (Figure 2).

## 4. Discussion

Over the past decade, a considerable number of studies evaluated the link of vitamin D deficiency, low PLT count, and altered PLT activation parameters with several diseases, i.e., coronary artery disease, diabetes, hypertension, venous thromboembolism, and altered bone remodeling due to OP [17,111]. However, despite vitamin D deficiency, altered PLT count, and/or related parameters also being associated with severe forms of SARS-CoV-2 infection, to date, no studies have investigated the potential association between vitamin D deficiency and PLT alteration. The main function of vitamin D is calcium metabolism with a role in bone structure, and its deficiency is linked with several pathological conditions, such as OP [2,23]. However, in addition to its *‘classical role’* in bone metabolism, vitamin D deficiency has a critical role in coagulation, inflammation, thrombosis, endothelial dysfunction, and immunological diseases [3,4,5,6,7]. Similarly, PLTs also perform a key role in coagulation, inflammation, thrombosis, endothelial dysfunction, immune response, and bone metabolism. Thus, in COVID-19 patients, the presence of a low levels of vitamin D associated with a low PLT count and a consequent alteration of several PLT activation parameters could represent a higher risk factor for developing a more critical form of the infection, which in turn could also lead to a higher risk of hypercoagulation, thrombosis, endothelial dysfunction, and altered bone remodeling [1,2,38,39,40,41,42]. A potential explanatory mechanism underlying the association of this menacing *duo* with COVID-19 infection could be represented by the fact that vitamin D deficiency leads to an increase in proinflammatory cytokine levels, such as TNF-α and IL-6, which enhance oxidative stress and, in turn, stimulate megakaryopoiesis, contributing to further PLT activation [45]. The induction of this event leads to the release of immature and activated PLTs from the bone marrow to the circulatory system, which increase and/or alter all parameters related to PLT activation, such as MPV and PDW. The increased release of cytokines in the presence of vitamin D deficiency may be associated with the VDR, a transcription factor that mediates the genomic effects of calcitriol, which is constitutively expressed in monocytes and macrophages. In addition, since vitamin D can decrease vascular cell adhesion molecule (VCAM)-1 and membrane type-1 matrix metalloproteinase (MT1-MMP) expression, thus preventing PLT activation and decreasing fibrinolysis and thrombosis [121], its deficiency also leads to further PLT activation and fibrinolytic activity. Thus, in patients with vitamin D deficiency, the combined effect of elevated TNF-α and IL-6 levels and increased release of adhesion molecules could lead to a further increase in PLT activation and aggregation. These alterations further reflect the hypercoagulable state present in severe COVID-19 patients, which could promote thromboembolic events [13]. Additionally, the associated effect of vitamin D deficiency, elevated TNF-α, and IL-6 with increased PLT activation and aggregation also plays a critical role in several aspects of osteoclastogenesis, enhancing the bone resorptive capacity via RANK/RANKL/OPG signaling, a system known for its roles in osteoclast maturation, as well as bone modeling and remodeling [122].

## 5. Conclusions

It is possible to speculate the presence of predisposing factors to bone fragility and OP in severe COVID-19 patients. Obviously, further potential risk factors for bone health in COVID-19 infection could be linked to aging, inflammation, metabolic diseases, kidney dysfunction, side-effects of some drugs such as glucocorticoids, and oxidative stress. This is just a hypothesis, and further studies are needed to validate our theory. It is plausible that the potential association between vitamin D deficiency and altered PLT-related parameters will emerge in the coming months; however, we think that the knowledge presented here should increase the sensitivity of clinicians caring for COVID-19 patients with altered vitamin D and PLT parameters, as well as highlight the need for more attention toward bone health status, while considering and stratifying patients for their comorbidities, age, and gender.

The different pervasive actions of vitamin D and PLTs on many organ systems and in several physiological and pathological conditions, such as coagulation, inflammation, thrombosis, endothelial dysfunction, immunological diseases, and bone remodeling, have given rise to many possible interactions between these factors and SARS-CoV-2 infection.

Although the data are far from conclusive in attributing a specific and clear role to the association between vitamin D and PLT in influencing the risk and outcome of COVID-19 disease, further research would be timely and revealing.

## Data Availability

The data presented in this study were extracted from the articles cited in the text.

## References

[1] Li Q., Guan X., Wu P., Wang X., Zhou L., Tong Y., Ren R., Leung K., Lau E., Wong J.Y. (2020). Early Transmission Dynamics in Wuhan, China, of Novel Coronavirus-Infected Pneumonia. N. Engl. J. Med..

[2] Richardson S., Hirsch J.S., Narasimhan M., Crawford J.M., McGinn T., Davidson K.W., Barnaby D.P., Becker L.B., Chelico J.D., Cohen S.L. (2020). Presenting Characteristics, Comorbidities, and Outcomes Among 5700 Patients Hospitalized With COVID-19 in the New York City Area. JAMA.

[3] Griffin D.O., Jensen A., Khan M., Chin J., Chin K., Saad J., Parnell R., Awwad C., Patel D. (2020). Pulmonary Embolism and Increased Levels of d-Dimer in Patients with Coronavirus Disease. Emerg. Infect. Dis..

[4] Spiezia L., Boscolo A., Poletto F., Cerruti L., Tiberio I., Campello E., Navalesi P., Simioni P. (2020). COVID-19-Related Severe Hypercoagulability in Patients Admitted to Intensive Care Unit for Acute Respiratory Failure. Thromb. Haemost..

[5] Helms J., Tacquard C., Severac F., Leonard-Lorant I., Ohana M., Delabranche X., Merdji H., Clere-Jehl R., Schenck M., CRICS TRIGGERSEP Group (Clinical Research in Intensive Care and Sepsis Trial Group for Global Evaluation and Research in Sepsis) (2020). High risk of thrombosis in patients with severe SARS-CoV-2 infection: A multicenter prospective cohort study. Intensiv. Care Med..

[6] Llitjos J., Leclerc M., Chochois C., Monsallier J., Ramakers M., Auvray M., Merouani K. (2020). High incidence of venous thromboembolic events in anticoagulated severe COVID-19 patients. J. Thromb. Haemost..

[7] Beyrouti R., Adams M.E., Benjamin L., Cohen H., Farmer S.F., Goh Y.Y., Humphries F., Jäger H.R., A Losseff N., Perry R.J. (2020). Characteristics of ischaemic stroke associated with COVID-19. J. Neurol. Neurosurg. Psychiatry.

[8] Lodigiani C., Iapichino G., Carenzo L., Cecconi M., Ferrazzi P., Sebastian T., Kucher N., Studt J.-D., Sacco C., Bertuzzi A. (2020). Venous and arterial thromboembolic complications in COVID-19 patients admitted to an academic hospital in Milan, Italy. Thromb. Res..

[9] Mitra A., Dwyre D.M., Schivo M., Iii G.R.T., Cohen S.H., Ku N., Graff J.P. (2020). Leukoerythroblastic reaction in a patient with COVID-19 infection. Am. J. Hematol..

[10] Wong J.E.L., Leo Y.S., Tan C.C. (2020). COVID-19 in Singapore—Current Experience. JAMA.

[11] Chen T., Wu D., Chen H., Yan W., Yang D., Chen G., Ma K., Xu D., Yu H., Wang H. (2020). Clinical characteristics of 113 deceased patients with coronavirus disease 2019: Retrospective study. BMJ.

[12] Buoro S., di Marco F., Rizzi M., Fabretti F., Lorini L.F., Cesa S., Fagiuoli S. (2020). Papa Giovanni XXIII Bergamo Hospital at the time of the COVID-19 outbreak: Letter from the warfront…. Int. J. Lab. Hematol..

[13] Tang N., Li D., Wang X., Sun Z. (2020). Abnormal coagulation parameters are associated with poor prognosis in patients with novel coronavirus pneumonia. J. Thromb. Haemost..

[14] Lippi G., Plebani M., Henry B.M. (2020). Thrombocytopenia is associated with severe coronavirus disease 2019 (COVID-19) infections: A meta-analysis. Clin. Chim. Acta.

[15] Chen G., Wu D., Guo W., Cao Y., Huang D., Wang H., Wang T., Zhang X., Chen H., Yu H. (2020). Clinical and immunological features of severe and moderate coronavirus disease 2019. J. Clin. Investig..

[16] Huang Y., Tu M., Wang S., Chen S., Zhou W., Chen D., Zhou L., Wang M., Zhao Y., Zeng W. (2020). Clinical characteristics of laboratory confirmed positive cases of SARS-CoV-2 infection in Wuhan, China: A retrospective single center analysis. Travel Med. Infect. Dis..

[17] Salamanna F., Maglio M., Landini M.P., Fini M. (2020). Platelet functions and activities as potential hematologic parameters related to Coronavirus Disease 2019 (Covid-19). Platelets.

[18] Merzon E., Tworowski D., Gorohovski A., Vinker S., Cohen A.G., Green I., Frenkel-Morgenstern M. (2020). Low plasma 25(OH) vitamin D level is associated with increased risk of COVID-19 infection: An Israeli population-based study. FEBS J..

[19] Hernández J.L., Nan D., Fernandez-Ayala M., García-Unzueta M., A Hernández-Hernández M., López-Hoyos M., Muñoz-Cacho P., Olmos J.M., Gutiérrez-Cuadra M., Ruiz-Cubillán J.J. (2020). Vitamin D Status in Hospitalized Patients with SARS-CoV-2 Infection. J. Clin. Endocrinol. Metab..

[20] Ilie P.C., Stefanescu S., Smith L. (2020). The role of vitamin D in the prevention of coronavirus disease 2019 infection and mortality. Aging Clin. Exp. Res..

[21] Endocrinology T.L.D. (2021). Vitamin D and COVID-19: Why the controversy?. Lancet Diabetes Endocrinol..

[22] Bennouar S., Cherif A.B., Kessira A., Bennouar D.-E., Abdi S. (2021). Vitamin D Deficiency and Low Serum Calcium as Predictors of Poor Prognosis in Patients with Severe COVID-19. J. Am. Coll. Nutr..

[23] Li Q., Cao Y., Chen L., Wu D., Yu J., Wang H., He W., Dong F., Chen W., Chen W. (2020). Hematological features of persons with COVID-19. Leukemia.

[24] National Health Commission of the People’s Republic of China (2020). The 5th trial version of Diagnosis and Treatment Scheme for Pneumonitis with 2019-nCoV Infection. http://www.nhc.gov.cn/yzygj/s7653p/202003/46c9294a7dfe4cef80dc7f5912eb1989shtml.

[25] Chen N., Zhou M., Dong X., Qu J., Gong F., Han Y., Qiu Y., Wang J., Liu Y., Wei Y. (2020). Epidemiological and clinical characteristics of 99 cases of 2019 novel coronavirus pneumonia in Wuhan, China: A descriptive study. Lancet.

[26] Kabak M., Çil B., Hocanlı I. (2021). Relationship between leukocyte, neutrophil, lymphocyte, platelet counts, and neutrophil to lymphocyte ratio and polymerase chain reaction positivity. Int. Immunopharmacol..

[27] Biino G., Santimone I., Minelli C., Sorice R., Frongia B., Traglia M., Ulivi S., Di Castelnuovo A., Gögele M., Nutile T. (2013). Age- and Sex-Related Variations in Platelet Count in Italy: A Proposal of Reference Ranges Based on 40987 Subjects’ Data. PLoS ONE.

[28] Balduini C.L., Noris P. (2014). Platelet count and aging. Haematologica.

[29] Zhang J., McCullough P.A., Tecson K.M. (2020). Vitamin D deficiency in association with endothelial dysfunction: Implications for patients with COVID-19. Rev. Cardiovasc. Med..

[30] Mohammad S., Mishra A., Ashraf M.Z. (2019). Emerging Role of Vitamin D and Its Associated Molecules in Pathways Related to Pathogenesis of Thrombosis. Biomolecules.

[31] Couldwell G., Machlus K.R. (2019). Modulation of megakaryopoiesis and platelet production during inflammation. Thromb. Res..

[32] Salamanna F., Maglio M., Sartori M., Tschon M., Fini M. (2020). Platelet Features and Derivatives in Osteoporosis: A Rational and Systematic Review on the Best Evidence. Int. J. Mol. Sci..

[33] Ringe J.D. (2020). Plain vitamin D or active vitamin D in the treatment of osteoporosis: Where do we stand today?. Arch. Osteoporos..

[34] Mazziotti G., Bilezikian J., Canalis E., Cocchi D., Giustina A. (2011). New understanding and treatments for osteoporosis. Endocrine.

[35] McLean R.R. (2009). Proinflammatory cytokines and osteoporosis. Curr. Osteoporos. Rep..

[36] Emkey G.R., Epstein S. (2014). Secondary osteoporosis: Pathophysiology & diagnosis. Best Pr. Res. Clin. Endocrinol. Metab..

[37] Canalis E., Mazziotti G., Giustina A., Bilezikian J.P. (2007). Glucocorticoid-induced osteoporosis: Pathophysiology and therapy. Osteoporos. Int..

[38] Napoli N., Elderkin A.L., Kiel D.P., Khosla S. (2020). Managing fragility fractures during the COVID-19 pandemic. Nat. Rev. Endocrinol..

[39] Conti P., Ronconi G., Caraffa A., Gallenga C., Ross R., Frydas I., Kritas S. (2020). Induction of pro-inflammatory cytokines (IL-1 and IL-6) and lung inflammation by Coronavirus-19 (COVI-19 or SARS-CoV-2): Anti-inflammatory strategies. J. Biol. Regul. Homeost. Agents.

[40] Di Filippo L., Formenti A.M., Rovere-Querini P., Carlucci M., Conte C., Ciceri F., Zangrillo A., Giustina A. (2020). Hypocalcemia is highly prevalent and predicts hospitalization in patients with COVID-19. Endocrine.

[41] Zhang S., Liu Y., Wang X., Yang L., Li H., Wang Y., Liu M., Zhao X., Xie Y., Yang Y. (2020). SARS-CoV-2 binds platelet ACE2 to enhance thrombosis in COVID-19. J. Hematol. Oncol..

[42] Marazuela M., Giustina A., Puig-Domingo M. (2020). Endocrine and metabolic aspects of the COVID-19 pandemic. Rev. Endocr. Metab. Disord..

[43] Obitsu S., Ahmed N., Nishitsuji H., Hasegawa A., Nakahama K.-I., Morita I., Nishigaki K., Hayashi T., Masuda T., Kannagi M. (2009). Potential enhancement of osteoclastogenesis by severe acute respiratory syndrome coronavirus 3a/X1 protein. Arch. Virol..

[44] Lanham-New S.A., Webb A.R., Cashman K.D., Buttriss J.L., Fallowfield J.L., Masud T., Hewison M., Mathers J.C., Kiely M., Welch A.A. (2020). Vitamin D and SARS-CoV-2 virus/COVID-19 disease. BMJ Nutr. Prev. Health.

[45] Schwalfenberg G. (2010). A review of the critical role of vitamin D in the functioning of the immune system and the clinical implications of vitamin D deficiency. Mol. Nutr. Food Res..

[46] Zemb P., Bergman P., Camargo C., Cavalier E., Cormier C., Courbebaisse M., Hollis B., Joulia F., Minisola S., Pilz S. (2020). Vitamin D deficiency and the COVID-19 pandemic. J. Glob. Antimicrob. Resist..

[47] Blondon M., Biver E., Braillard O., Righini M., Fontana P., Casini A. (2019). Thrombin generation and fibrin clot structure after vitamin D supplementation. Endocr. Connect..

[48] Kaufman H.W., Niles J.K., Kroll M.H., Bi C., Holick M.F. (2020). SARS-CoV-2 positivity rates associated with circulating 25-hydroxyvitamin D levels. PLoS ONE.

[49] Maghbooli Z., Sahraian M.A., Ebrahimi M., Pazoki M., Kafan S., Tabriz H.M., Hadadi A., Montazeri M., Nasiri M., Shirvani A. (2020). Vitamin D sufficiency, a serum 25-hydroxyvitamin D at least 30 ng/mL reduced risk for adverse clinical outcomes in patients with COVID-19 infection. PLoS ONE.

[50] Ali N. (2020). Role of vitamin D in preventing of COVID-19 infection, progression and severity. J. Infect. Public Health.

[51] Lips P., Cashman K.D., Lamberg-Allardt C., Bischoff-Ferrari H., Obermayer-Pietsch B., Bianchi M.L., Stepan J., Fuleihan G.E.-H., Bouillon R. (2019). Current vitamin D status in European and Middle East countries and strategies to prevent vitamin D deficiency: A position statement of the European Calcified Tissue Society. Eur. J. Endocrinol..

[52] Pagano M.T., Peruzzu D., Ruggieri A., Ortona E., Gagliardi M.C. (2020). Vitamin D and Sex Differences in COVID-19. Front. Endocrinol..

[53] Benskin L.L. (2020). A Basic Review of the Preliminary Evidence That COVID-19 Risk and Severity Is Increased in Vitamin D Deficiency. Front. Public Health.

[54] Mansur J.L. (2020). Letter: Low population mortality from COVID-19 in countries south of latitude 35 degrees North supports vitamin D as a factor determining severity. Aliment. Pharmacol. Ther..

[55] Bilezikian J.P., Bikle D., Hewison M., Lazaretti-Castro M., Formenti A.M., Gupta A., Madhavan M.V., Nair N., Babalyan V., Hutchings N. (2020). MECHANISMS IN ENDOCRINOLOGY: Vitamin D and COVID-19. Eur. J. Endocrinol..

[56] Martineau A.R., Jolliffe D.A., Greenberg L., Aloia J.F., Bergman P., Dubnov-Raz G., Esposito S., Ganmaa D., Ginde A.A., Goodall E.C. (2019). Vitamin D supplementation to prevent acute respiratory infections: Individual participant data meta-analysis. Heal. Technol. Assess..

[57] Ahmed F. (2020). A Network-Based Analysis Reveals the Mechanism Underlying Vitamin D in Suppressing Cytokine Storm and Virus in SARS-CoV-2 Infection. Front. Immunol..

[58] Chakhtoura M., Napoli N., Fuleihan G.E.H. (2020). Commentary: Myths and facts on vitamin D amidst the COVID-19 pandemic. Metabolism.

[59] Kumar R., Rathi H., Haq A., Wimalawansa S.J., Sharma A. (2020). Putative roles of vitamin D in modulating immune response and immunopathology associated with COVID-19. Virus Res..

[60] Chandran M., Maung A.C., Mithal A., Parameswaran R. (2020). Vitamin D in COVID-19: Dousing the fire or averting the storm?—A perspective from the Asia-Pacific. Osteoporos. Sarcopenia.

[61] Lewiecki E.M. (2020). Vitamin D and COVID-19: Is something better than nothing?. Osteoporos. Sarcopenia.

[62] Mitchell F. (2020). Vitamin-D and COVID-19: Do deficient risk a poorer outcome?. Lancet Diabetes Endocrinol..

[63] Mohan M., Cherian J.J., Sharma A. (2020). Exploring links between vitamin D deficiency and COVID-19. PLOS Pathog..

[64] Smolders J., Ouweland J.V.D., Geven C., Pickkers P., Kox M. (2020). Letter to the Editor: Vitamin D deficiency in COVID-19: Mixing up cause and consequence. Metabolism.

[65] Tramontana F., Napoli N., Fuleihan G.E.-H., Strollo R. (2020). The D-side of COVID-19: Musculoskeletal benefits of vitamin D and beyond. Endocrine.

[66] Salvio G., Gianfelice C., Firmani F., Lunetti S., Balercia G., Giacchetti G. (2020). Bone Metabolism in SARS-CoV-2 Disease: Possible Osteoimmunology and Gender Implications. Clin. Rev. Bone Miner. Metab..

[67] Al-Daghri N.M., Yakout S., Aljohani N., Al-Saleh Y., Al-Attas O.S., McTernan P.G., Alokail M.S. (2017). Changes in serum cytokines and vitamin D in Saudi postmenopausal women with osteoporosis. Int. J. Clin. Exp. Med..

[68] Laird E., Ward M., McSorley E., Strain J., Wallace J. (2010). Vitamin D and Bone Health; Potential Mechanisms. Nutrients.

[69] Blanch-Rubió J., Soldevila-Domenech N., Tío L., Llorente-Onaindia J., Ciria-Recasens M., Polino L., Gurt A., De La Torre R., Maldonado R., Monfort J. (2020). Influence of anti-osteoporosis treatments on the incidence of COVID-19 in patients with non-inflammatory rheumatic conditions. Aging.

[70] Rinonapoli G., Ruggiero C., Meccariello L., Bisaccia M., Ceccarini P., Caraffa A. (2021). Osteoporosis in Men: A Review of an Underestimated Bone Condition. Int. J. Mol. Sci..

[71] Gravholt C.H., Viuff M.H., Brun S., Stochholm K., Andersen N.H. (2019). Turner syndrome: Mechanisms and management. Nat. Rev. Endocrinol..

[72] Meftahi G.H., Jangravi Z., Sahraei H., Bahari Z. (2020). The possible pathophysiology mechanism of cytokine storm in elderly adults with COVID-19 infection: The contribution of “inflame-aging”. Inflamm. Res..

[73] Michelson A.D. (2013). Platelets. London.

[74] Morrell C.N., Aggrey A.A., Chapman L.M., Modjeski K.L. (2014). Emerging roles for platelets as immune and inflammatory cells. Blood.

[75] Semple J.W., Italiano J.E., Freedman J. (2011). Platelets and the immune continuum. Nat. Rev. Immunol..

[76] Koupenova M., Kehrel B.E., Corkrey H.A., Freedman J.E. (2016). Thrombosis and platelets: An update. Eur. Hear. J..

[77] Koupenova M., Clancy L., Corkrey H.A., Freedman J.E. (2018). Circulating Platelets as Mediators of Immunity, Inflammation, and Thrombosis. Circ. Res..

[78] Etulain J., Schattner M. (2014). Glycobiology of platelet-endothelial cell interactions. Glycobiology.

[79] Wagner D.D., Frenette P.S. (2008). The vessel wall and its interactions. Blood.

[80] Yeaman M.R. (2009). Platelets in defense against bacterial pathogens. Cell. Mol. Life Sci..

[81] Assinger A. (2014). Platelets and Infection—An Emerging Role of Platelets in Viral Infection. Front. Immunol..

[82] Vanderschueren S., De Weerdt A., Malbrain M., Vankersschaever D., Frans E., Wilmer A., Bobbaers H. (2000). Thrombocytopenia and prognosis in intensive care. Crit. Care Med..

[83] Moll M., Zon R.L., Sylvester K.W., Chen E.C., Cheng V., Connell N., Fredenburgh L.E., Baron R.M., Cho M.H., Woolley A.E. (2020). VTE in ICU Patients With COVID-19. Chest.

[84] Klok F., Kruip M., van der Meer N., Arbous M., Gommers D., Kant K., Kaptein F., van Paassen J., Stals M., Huisman M. (2020). Incidence of thrombotic complications in critically ill ICU patients with COVID-19. Thromb. Res..

[85] Ackermann M., Verleden S., Kuehnel M., Haverich A., Welte T., Laenger F., Vanstapel A., Werlein C., Stark H., Tzankov A. (2020). Pulmonary Vascular Endothelialitis, Thrombosis, and Angiogenesis in Covid-19. N. Engl. J. Med..

[86] Rapkiewicz A.V., Mai X., Carsons S.E., Pittaluga S., Kleiner D.E., Berger J.S., Thomas S., Adler N., Charytan D., Gasmi B. (2020). Megakaryocytes and platelet-fibrin thrombi characterize multi-organ thrombosis at autopsy in COVID-19: A case series. EClinicalMedicine.

[87] Manne B.K., Denorme F., Middleton E.A., Portier I., Rowley J.W., Stubben C.J., Petrey A.C., Tolley N.D., Guo L., Cody M.J. (2020). Platelet gene expression and function in patients with COVID-19. Blood.

[88] Hottz E.D., Azevedo-Quintanilha I.G., Palhinha L., Teixeira L., Barreto E.A., Pão C.R.R., Righy C., Franco S., Souza T.M.L., Kurtz P. (2020). Platelet activation and platelet-monocyte aggregate formation trigger tissue factor expression in patients with severe COVID-19. Blood.

[89] Comer S.P., Cullivan S., Szklanna P.B., Weiss L., Cullen S., Kelliher S., Smolenski A., Murphy C., Altaie H., Curran J. (2021). COVID-19 induces a hyperactive phenotype in circulating platelets. PLoS Biol..

[90] Zaid Y., Puhm F., Allaeys I., Naya A., Oudghiri M., Khalki L., Limami Y., Zaid N., Sadki K., Ben El Haj R. (2020). Platelets Can Associate With SARS-CoV-2 RNA and Are Hyperactivated in COVID-19. Circ. Res..

[91] Rahman A., Niloofa R., Jayarajah U., De Mel S., Abeysuriya V., Seneviratne S.L. (2021). Hematological Abnormalities in COVID-19: A Narrative Review. Am. J. Trop. Med. Hyg..

[92] Qu R., Ling Y., Zhang Y., Wei L., Chen X., Li X., Liu X., Liu H., Guo Z., Ren H. (2020). Platelet-to-lymphocyte ratio is associated with prognosis in patients with coronavirus disease-19. J. Med. Virol..

[93] Yang A.-P., Liu J.-P., Tao W.-Q., Li H.-M. (2020). The diagnostic and predictive role of NLR, d-NLR and PLR in COVID-19 patients. Int. Immunopharmacol..

[94] Chan A.S., Rout A. (2020). Use of Neutrophil-to-Lymphocyte and Platelet-to-Lymphocyte Ratios in COVID-19. J. Clin. Med. Res..

[95] Alnor A., Sandberg M.B., Toftanes B.E., Vinholt P.J. (2021). Platelet parameters and leukocyte morphology is altered in COVID-19 patients compared to non-COVID-19 patients with similar symptomatology. Scand. J. Clin. Lab. Investig..

[96] Liu Y., Sun W., Guo Y., Chen L., Zhang L., Zhao S., Long D., Yu L. (2020). Association between platelet parameters and mortality in coronavirus disease 2019: Retrospective cohort study. Platelets.

[97] Güçlü E., Kocayiğit H., Okan H.D., Erkorkmaz U., Yürümez Y., Yaylacı S., Koroglu M., Uzun C., Karabay O. (2020). Effect of COVID-19 on platelet count and its indices. Rev. Assoc. Méd. Bras..

[98] Ozenen G.G., Bal Z.S., Umit Z., Bilen N.M., Arslan S.Y., Yurtseven A., Saz E.U., Burcu B., Sertoz R., Kurugol Z. (2021). Demographic, clinical, and laboratory features of COVID-19 in children: The role of mean platelet volume in predicting hospitalization and severity. J. Med. Virol..

[99] Wool G.D., Miller J.L. (2020). The Impact of COVID-19 Disease on Platelets and Coagulation. Pathobiology.

[100] Hille L., Lenz M., Vlachos A., Grüning B., Hein L., Neumann F., Nührenberg T.G., Trenk D. (2020). Ultrastructural, transcriptional, and functional differences between human reticulated and non-reticulated platelets. J. Thromb. Haemost..

[101] Yang M., Ng M.H., Li C.K. (2005). Thrombocytopenia in patients with severe acute respiratory syndrome (review). Hematology.

[102] Jolicoeur P., Lamontagne L. (1995). Impairment of Bone Marrow Pre-B and B Cells in MHV3 Chronically-Infected Mice. Corona-and Related Viruses.

[103] World Health Organization Report of the WHO-China Joint Mission on Coronavirus Disease 2019 (COVID-19). Geneva, 2020. https://www.who.int/docs/default-source/coronaviruse/who-china-joint-mission-on-covid-19-final-report.pdf.

[104] Zhang W., Zhao Y., Zhang F., Wang Q., Li T., Liu Z., Wang J., Qin Y., Zhang X., Yan X. (2020). The use of anti-inflammatory drugs in the treatment of people with severe coronavirus disease 2019 (COVID-19): The Perspectives of clinical immunologists from China. Clin. Immunol..

[105] Li X.-S., Zhang J.-R., Meng S.-Y., Li Y., Wang R.-T. (2012). Mean platelet volume is negatively associated with bone mineral density in postmenopausal women. J. Bone Miner. Metab..

[106] Aypak C., Türedi Ö., Bircan M.A., Civelek G.M., Araz M. (2016). Association between mean platelet volume and bone mineral density in postmenopausal women. J. Phys. Ther. Sci..

[107] Vural M., Mert M., Erhan B., Gunduz B., Keles B.Y., Erdem A.E., Bozan A., Arslan H. (2017). Is there any relationship between mean platelet volume, bone mineral density and vitamin d in postmenopausal women?. Acta Med. Mediterr..

[108] Kim H.-L., Cho H.Y., Park I.Y., Choi J.M., Kim M., Jang H.J., Hwang S.-M. (2011). The Positive Association between Peripheral Blood Cell Counts and Bone Mineral Density in Postmenopausal Women. Yonsei Med. J..

[109] Eroglu S., Karatas G. (2019). Platelet/lymphocyte ratio is an independent predictor for osteoporosis. Saudi Med. J..

[110] Koseoglu S.B. (2017). Bone loss & platelet-to-lymphocyte ratio. Biomark. Med..

[111] Kristjansdottir H., Mellström D., Johansson P., Karlsson M., Vandenput L., Lorentzon M., Herlitz H., Ohlsson C., Lerner U., Lewerin C. (2020). High platelet count is associated with low bone mineral density: The MrOS Sweden cohort. Osteoporos. Int..

[112] Lippi G., Meschi T., Borghi L. (2012). Mean platelet volume increases with aging in a large population study. Thromb. Res..

[113] Ciovacco W.A., Cheng Y.-H., Horowitz M.C., Kacena M.A. (2009). Immature and mature megakaryocytes enhance osteoblast proliferation and inhibit osteoclast formation. J. Cell. Biochem..

[114] Beeton C., Bord S., Ireland D., Compston J. (2006). Osteoclast formation and bone resorption are inhibited by megakaryocytes. Bone.

[115] Föger-Samwald U., Dovjak P., Azizi-Semrad U., Kerschan-Schindl K., Pietschmann P. (2020). Osteoporosis: Pathophysiology and therapeutic options. EXCLI J..

[116] Zheng K., Zhang W.C., Xu Y.Z., Geng D.C. (2020). COVID-19 and the bone: Underestimated to consider. Eur. Rev. Med. Pharmacol. Sci..

[117] Okamoto K., Nakashima T., Shinohara M., Negishi-Koga T., Komatsu N., Terashima A., Sawa S., Nitta T., Takayanagi H. (2017). Osteoimmunology: The Conceptual Framework Unifying the Immune and Skeletal Systems. Physiol. Rev..

[118] Dewitte A., Tanga A., Villeneuve J., Lepreux S., Ouattara A., Desmoulière A., Combe C., Ripoche J. (2015). New frontiers for platelet CD154. Exp. Hematol. Oncol..

[119] Sowa J.M., Crist S.A., Ratliff T.L., Elzey B.D. (2009). Platelet influence on T- and B-cell responses. Arch. Immunol. Ther. Exp..

[120] Breart G., Cooper C., Meyer O., Speirs C., Deltour N., Reginster J.Y. (2009). Osteoporosis and venous thromboembolism: A retrospective cohort study in the UK General Practice Research Database. Osteoporos. Int..

[121] Sorenson M., Grant W.B. (2012). Does vitamin D deficiency contribute to erectile dysfunction?. Dermatoendocrinology.

[122] Tobeiha M., Moghadasian M.H., Amin N., Jafarnejad S. (2020). RANKL/RANK/OPG Pathway: A Mechanism Involved in Exercise-Induced Bone Remodeling. Biomed Res. Int..

